# Niveles séricos de antígeno prostático específico (PSA) tras vacunación primaria con BNT162b2

**DOI:** 10.1515/almed-2023-0120

**Published:** 2023-09-15

**Authors:** Simone De Nitto, Laura Pighi, Gian Luca Salvagno, Giuseppe Lippi

**Affiliations:** Service of Laboratory Medicine, Pederzoli Hospital, Verona, Italia; Section of Clinical Biochemistry and School of Medicine, University of Verona, Verona, Italia

**Keywords:** COVID-19, SARS-CoV-2, PSA, próstata

**Figura 1:**
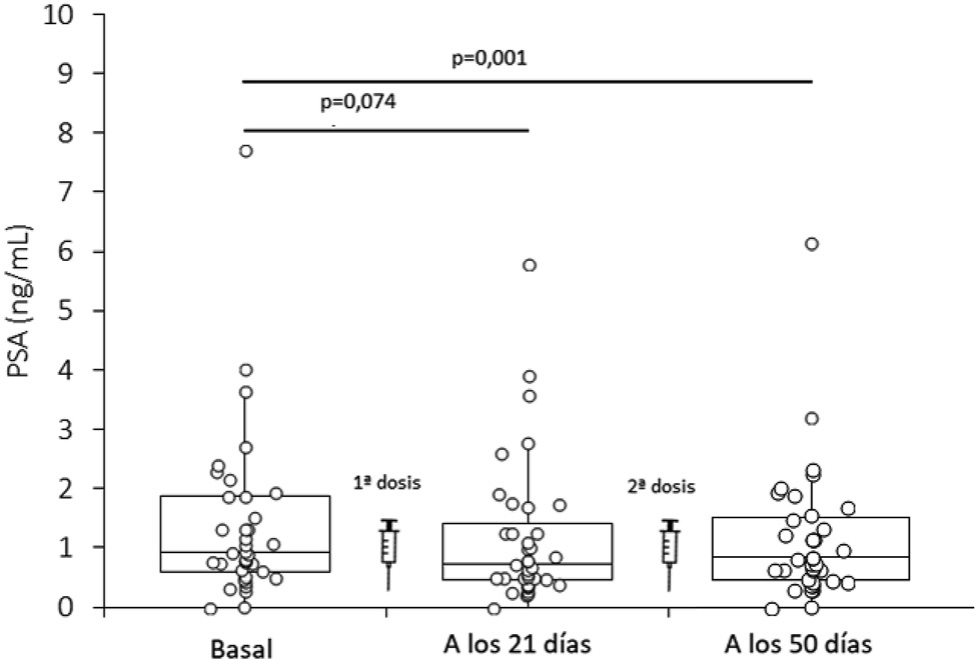
Variación del antígeno prostático específico (PSA) en receptores claramente sanos de un ciclo de vacunación primaria con la vacuna BNT162b2. PSA, antígeno prostático específico.

Estimado Editor,

Recientemente, se ha aportado evidencia de que los niveles séricos del antígeno prostático específico (PSA) podrían aumentar significativamente con respecto a los niveles basales, en receptores de la vacuna BNT162b2 (Pfizer-BioNTech, Mainz, Alemania) contra la enfermedad por coronavirus 2019 (COVID-19) [1]. Con el fin de indagar en este intrigante fenómeno, realizamos un análisis retrospectivo de 37 trabajadores sanos (edad media: 61 ± 8 años) del hospital Pederzoli de Peschiera del Garda (Verona, Italia), que completaron el ciclo de vacunación primaria con la vacuna BNT162b2 contra la COVID-19. Se tomaron muestras sanguíneas mediante venopunción estándar inmediatamente antes de la primera dosis de BNT162b2, a los 21 días, e inmediatamente antes de la segunda dosis de BNT162b2, y transcurrido un mes desde la administración de la segunda dosis de la vacuna (esto es, 50 días después de la primera dosis de BNT162b2), tal como se muestra en la Figura 1. El protocolo del estudio ha sido descrito anteriormente [2]. Se midieron los niveles séricos de PSA en un analizador Roche Cobas e801 (Roche Diagnostics, Basel, Suiza; intervalo de medición: 0,006–100 ng/mL; imprecisión total: 1,5–5,1 %). Los resultados de las mediciones realizadas en tres ocasiones se presentan como medias y rangos intercuartílicos. Las diferencias se analizaron mediante la prueba U de Mann-Whitney (Analyse-it Software Ltd, Leeds, Reino Unido). Todos los participantes del estudio firmaron el consentimiento informado. El presente estudio se realizó de conformidad con los principios de la Declaración de Helsinki y fue aprobado por el Comité de Ética de las Provincias de Verona y Rovigo (59COVIDCESC; 8 de noviembre de 2021).

En la Figura 1 se muestran los resultados de este estudio. Los niveles séricos de PSA no variaron significativamente con respecto a los valores basales (mediana: 0,9 ng/mL; RIC: 0,61–1,88 ng/mL) tras la primera dosis de BNT162b2 (mediana: 0,7 ng/mL; RIC: 0,48–1,26 ng/mL; p=0,074), e incluso disminuyeron con respecto a los niveles basales tras la segunda dosis de BNT162b2 (mediana: 0,7 ng/mL; RIC: 0,39–1,29 ng/mL; p=0,001).

En términos generales, nuestros resultados no confirman la evidencia publicada anteriormente, de que los niveles séricos de PSA podrían aumentar tras la completar el ciclo de vacunación primaria con la vacuna BNT162b2. De hecho, observamos que la vacuna BNT162b2 podría tener un efecto beneficioso en la biología y función de la próstata, al reducir los niveles circulantes de PSA. Concretamente, cuestionamos que las variaciones en los niveles de PSA observados por Frumer y col [1] debieran ser considerados clínicamente significativos. Así, los autores comunicaron un incremento de 0,03 (RIC: −0,12 a 0,28) y 0,09 (RIC: −0,05 a 0,34) ng/dL tras la primera y la tercera dosis de la vacuna, respectivamente. Ambos valores tienen una baja significación clínica, ya que la mediana de la diferencia crítica de este biomarcador es del 20,5 % [3], que se encuentra varios órdenes de magnitud por encima de la variación documentada por Frumer y col [1].
